# The transfer of multigene panel testing for hereditary breast and ovarian cancer to healthcare: What are the implications for the management of patients and families?

**DOI:** 10.18632/oncotarget.12699

**Published:** 2016-10-15

**Authors:** Marie Eliade, Jeremy Skrzypski, Amandine Baurand, Caroline Jacquot, Geoffrey Bertolone, Catherine Loustalot, Charles Coutant, France Guy, Pierre Fumoleau, Yannis Duffourd, Laurent Arnould, Alexandra Delignette, Marie-Martine Padéano, Côme Lepage, Géraldine Raichon-Patru, Axelle Boudrant, Marie-Christine Bône-Lépinoy, Anne-Laure Villing, Aurélie Charpin, Karine Peignaux, Sandy Chevrier, Frédérique Vegran, François Ghiringhelli, Romain Boidot, Nicolas Sevenet, Sarab Lizard, Laurence Faivre

**Affiliations:** ^1^ Centre of Genetic, Children Hospital, CHU, Dijon, France; ^2^ Oncogenetic Unit, Centre Georges-François Leclerc Centre, Dijon, France; ^3^ Gynecological Surgery, Georges-François Leclerc Centre, Dijon, France; ^4^ Radiology unit, Georges-François Leclerc Centre, Dijon, France; ^5^ Medical Oncology, Georges-François Leclerc Centre, Dijon, France; ^6^ Orphanomix, Dijon, France; ^7^ Biology and Tumor Pathology Department, Georges-François Leclerc Centre, Dijon, France; ^8^ Elithis Tower, Radiology, Dijon, France; ^9^ Hepato-Gastroenterology and Digestive Oncology, François Mitterand Hospital, CHU, Dijon, France; ^10^ Oncology, Les Chanaux Hospital, Macon, France; ^11^ Oncology, William Morey Hospital, Chalon-sur-Saône, France; ^12^ Oncology, Drevon Clinic, Dijon, France; ^13^ Oncology, Hospital, Auxerre, France; ^14^ Radiotherapy Unit, Georges-François Leclerc Centre, Dijon, France; ^15^ Platform of Transfer in Cancer Biology, Georges-François Leclerc Centre, Dijon, France; ^16^ Bergonié Institute, Bordeaux, France; ^17^ Burgundy Franche-Comté University, INSERM LNC UMR866, Dijon, France; ^18^ Burgundy Franche-Comté University, Dijon, France

**Keywords:** next generation sequencing, breast and ovarian cancer susceptibility genes, genomic capture, candidate genes, management

## Abstract

Until recently, the molecular diagnosis of hereditary breast and ovarian cancer (HBOC) was mostly based on *BRCA1/2* testing. Next generation sequencing and the recent discovery of new genes involved in HBOC now permit the transfer of genomic capture targeting multiple candidate genes from research to clinical use. However, the implications for the management of patients and their families have not been extensively studied, in particular since some of these genes are not well-established cancer predisposing genes. We studied 583 consecutive patients from Burgundy (France) fulfilling the criteria for BRCA testing using a next generation sequencing 25-genes panel including 20 well-established high-risk cancer genes as well as more recently identified predisposing HBOC cancer. A pathogenic *BRCA1/2* mutation was found in 51 patients (9%). Besides, we found 37 pathogenic or likely pathogenic mutations in 10 different high to low-risk genes in 34 patients (6%). The most frequently mutated genes were *CHEK2* (*n* = 12; 2%), *ATM* (*n* = 9; 1.5%), and *PALB2* (*n* = 4; 0.6%). Three patients had a mutation in two different predisposing genes. The analysis of clinical actionability conducted in mutation-positive individuals revealed that additional disease-specific screening and/or prevention measures beyond those based on personal and family history alone had been recommended in 69% of cases. In conclusion, multigene panel testing is a powerful tool to identifying high to low-risk HBOC susceptibility genes. The penetrance and spectrum of cancers with these other genes are sometimes undefined, and further collaborative work is crucial to address this question.

## INTRODUCTION

Hereditary breast and ovarian cancer (HBOC) is characterized by the early onset of breast (BC) and/or ovarian cancer (OC), multiple primaries, bilateral tumors and a family history of cancer of the same spectrum in relatives. About 5-7% of BC and 20-25% of OC are thought to be due to rare variants conferring a hereditary predisposition to cancer [1, 2]. The penetrance of these rare variants may be highly variable depending on the gene considered. The use of multigene panels raises questions about the definition of risk [3]. *BRCA1* and *BRCA2,* the first gene mutations associated with a predisposition for a high risk of breast cancer*,* confer an estimated risk of 65 and 45% for BC and 39 and 10% for OC, respectively [4]. *BRCA1/2* mutations are responsible for 30% of early-onset breast cancers and 90% of family histories of BC and OC [5-8]. Genetic testing and management guidelines are now widely available. Mutations in other highly penetrant genes, such as *PTEN*, *CDH1*, *STK11* and *TP53*, can cause cancer susceptibility syndromes [9-12], but remain rare. *MLH1, MSH2, MSH6* and *PMS2* also contribute to a hereditary risk of OC. The criteria for genetic testing are specific to these predispositions, according to their disease spectrum.

In the past few years, since the arrival of next generation sequencing (NGS), several predisposing genes, mainly of moderate penetrance, have been discovered, thus explaining additional HBOC pedigrees. The majority of these genes are part of the DNA repair/*BRCA* pathway [13-16]. NGS has made it possible for clinicians to order a single test that evaluates multiple genes simultaneously in a cost-effective and efficient fashion, thus enabling a more complete genetic evaluation [17]. In the last 2 years, the literature assessing the contribution of multigene panel testing in cohorts of patients with HBOC has grown. The results have been consistent even though the inclusion criteria sometimes differ [17-30]. The next challenge is to determine the consequences of these results on clinical management [NCCN clinical practice Guidelines-see URL]. High-penetrance gene mutations have a high well-defined cancer risk by site; actionability for these genes is high, and evidence-based national guidelines exist for risk reduction in at least one organ system; the implications for other family members are thus straightforward. Moderate-penetrance gene mutations carry a more moderate risk, and organ-specific cancer risks are fairly well-defined for at least one cancer site; actionability for these genes is moderate, though there is enough evidence to go beyond empiric risk and to justify enhanced surveillance in the proband for at least one at-risk site. However, the implications for other family members may not be as straightforward. Low-penetrance gene mutations have a lower or uncertain risk and the organ-specific cancer risks are vague; actionability is low, due to the lack of established evidence-based guidelines; screening and management recommendations depend on empiric risk estimates and case reports in the literature. The implications for other family members are not well defined. In 2015, the National Comprehensive Cancer Network (NCCN) in the USA published guidelines for high-penetrance gene mutations as well as a number of moderate penetrance gene mutations, updated in 2016 [see URL]. These included *PALB2, CHEK2* and *ATM* pathogenic or probably pathogenic variants .

The aim of this study was to determine, in a new cohort of 583 patients with HBOC from the French region of Burgundy, the clinical actionability of variants found using a multigene panel for HBOC. Actionability concerned different options, including the modification of cancer surveillance, specific risk-reduction measures, treatment guidance, customized gene-specific treatment options, and the identification of at-risk family members.

## RESULTS

### Mutations identified in the cohort

**Figure 1:**
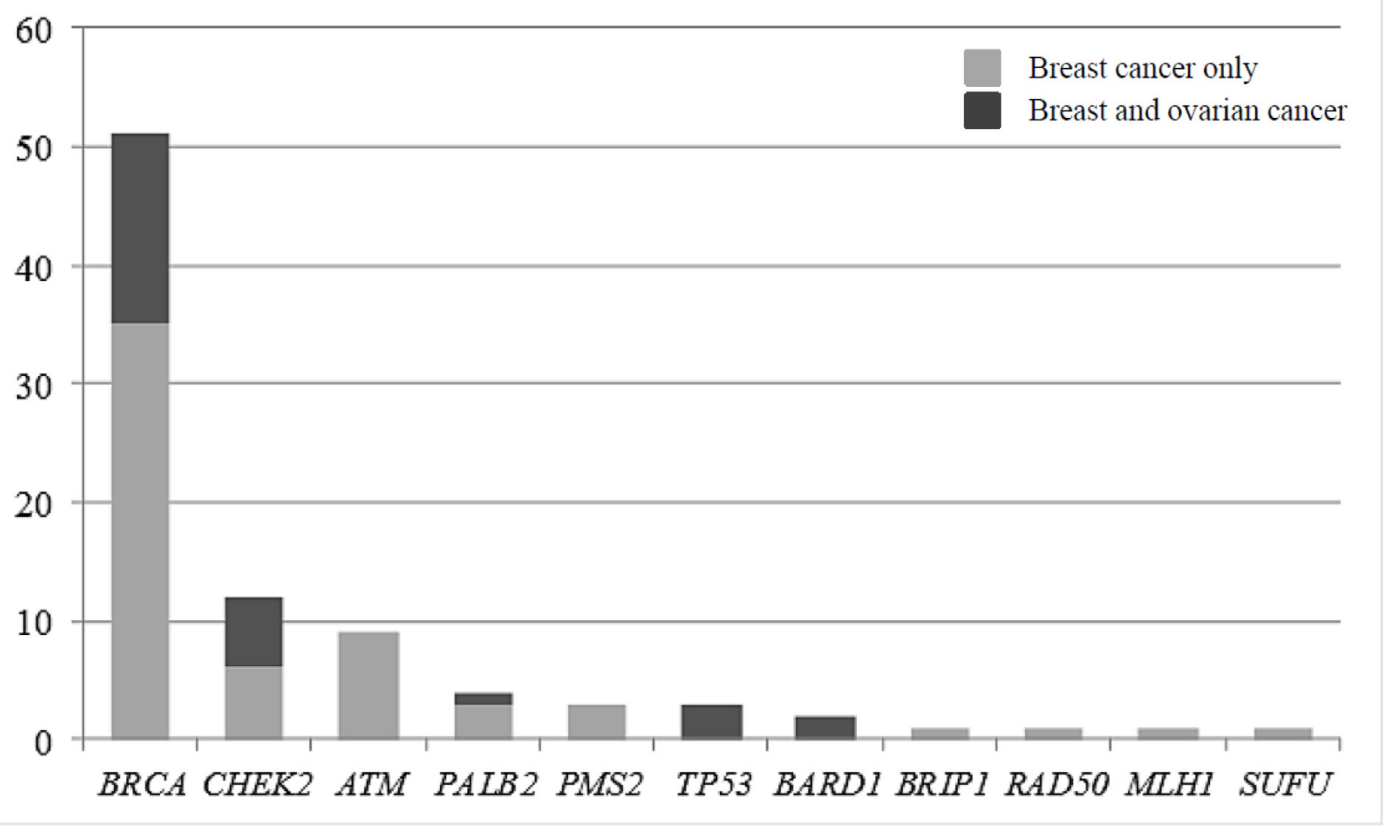
Distribution of pathogenic and likely-pathogenic mutations detected in genes other than *BRCA* according to cancer presentation in the patient and family In light gray, families with breast cancer presentation, and in dark grey, families with at least one patient with an ovarian cancer.

**Figure 2:**
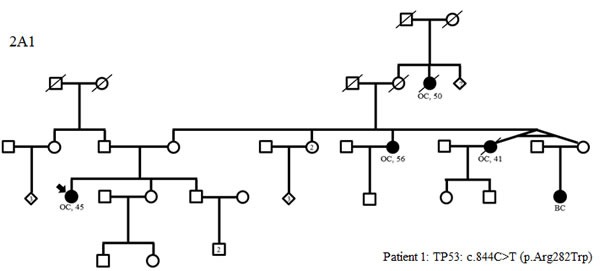

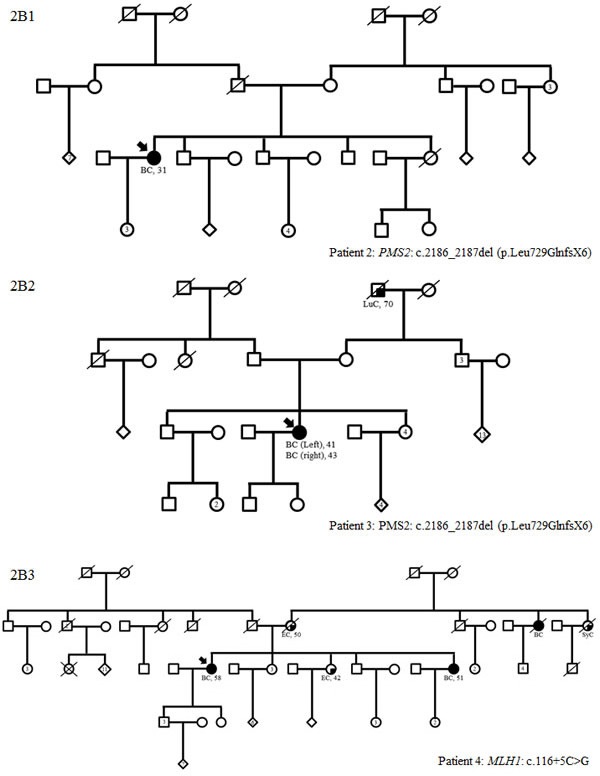

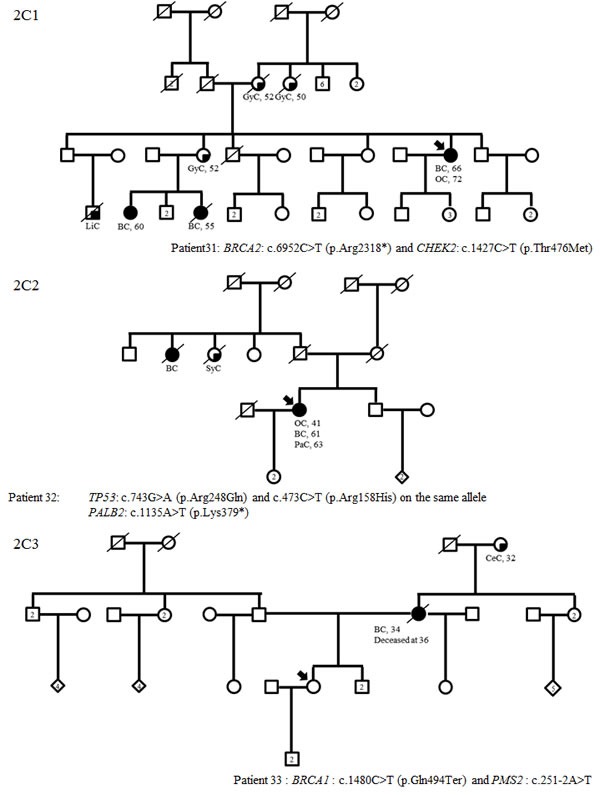
Family trees in patients with *TP53* (A1), MMR gene mutations (B1-3) and variants in two predisposing genes (C1-3) (possibly including a *TP53* or MMR mutation) BC: breast cancer; OC: ovarian cancer, LiC: liver cancer, GyC: gynecological cancer, SyC: systemic cancer, PaC: pancreatic cancer, CeC: cervical cancer, PrC: prostate cancer, CRC: colorectal cancer, TeC: testis cancer, HoD: Hodgkin's disease, EC: endometrial cancer, Me: melanoma, LuC: lung cancer

Of the 583 subjects tested, a pathogenic or probably pathogenic *BRCA1/2* mutation was found in 51 (9%) patients. Besides, thirty-seven pathogenic or probably pathogenic variants were found in 10 other genes of the panel. The distribution of mutations and their clinical presentation are given in Figure 1 and Table 1A and 1B. Six mutations were found in high-penetrance genes, with no diagnostic criteria that could have guided traditional genetic testing in the majority of them. It included two *TP53* pedigrees that did not fulfill Chompret criteria (Figure 2A_1_ and 2C_1_); four MMR mutations, in two patients with single-affected early-onset BC, one patient with familial BC, and one patient with BC but a family history of two endometrial cancers, which could have led to MMR testing in the relatives (Figure 2B_1_, B_2_, B_3_, C_3_), with a negative search for microsatellite instability in three breast tumors with available material. Twenty-one mutations were found in moderate-risk HBOC genes. The most frequently mutated genes were *CHEK2* (*n* = 12; 2% of the total cohort, 13% of the positive cohort), *ATM* (*n* = 9; 1.5% of the total cohort, 10% of the positive cohort) and *PALB2* (*n* = 4; 0.6% of the total cohort, 4.5% of the positive cohort), mostly in families with BC only. Other results included two *BARD1* mutations in families with BC and OC, one *BRIP1* and one *RAD50* mutation in families with BC only (Figure 1, Table 1). Three patients with a mutation in two different genes were found. One patient had pathogenic mutations in both *TP53* and *PALB2*, one had deleterious mutations in both *BRCA2* and *CHEK2* and another one in both *BRCA1* and *PMS2* (Figure 2C_1_, C_2_ and C_3_). One probably pathogenic mutation in *SUFU* was detected and considered incidental given the absence of a personal or family history of medulloblastoma, and the absence of BC in the spectrum of *SUFU*-predisposing cancers to date. The index case presented BC at age 49, multiple cancers in her mother at age 69 (OC, endometrial and colorectal cancer) and liver cancer in her maternal grandfather at age 58. The majority of the findings were relevant to the clinical history (34/38, 89%). In addition, seven monoallelic mutations of *MUTYH* were found, which is the number expected in the general population, and 245 VUS were identified.

**Table 1A:** Characteristics of patients with pathogenic or probably pathogenic variants in genes other than *BRCA*

Patient Number High risk gene 1 2 3 4 Moderate to low –risk gene 5 6 7 8 9 10 11 12 13 14 15 16 17 18 19 20 21 22 23 24 25 26 27 28 29 30 Mutations in two different genes in the same patient 31 32 33 Incidental Finding 34	Gender Female Female Female Female Female Female Female Female Female Female Female Female Female Female Female Female Female Female Female Female Male Female Female Female Female Female Female Female Female Female Female Female Female Female	Gene *TP53* *PMS2* *PMS2* *MLH1* *CHEK2* *CHEK2* *CHEK2* *CHEK2* *CHEK2* *CHEK2* *CHEK2* *CHEK2* *CHEK2* *CHEK2* *ATM* *ATM* *ATM* *ATM* *ATM* *ATM* *ATM* *ATM* *ATM* *PALB2* *PALB2* *PALB2* *BARD1* *BARD1* *BRIP1* *RAD50* *CHEK2* *BRCA2* *PALB2* *TP53* (same allele) *PMS2* *BRCA1* *SUFU*	Variant c.844C>T (p.Arg282Trp) c.2186_2187del (p.Leu729GlnfsX6) c.2186_2187del (p.Leu729GlnfsX6) c.116+5G>C c.190G>A (p.Glu64Lys) c.1427C>T (p.Thr476Met) c.686del (p.Asn229IlefsX8) c.1312G>T (p.Asp438Tyr) c.349A>G (p.Arg117Gly) c.1229del (p.Thr410MetfsX15) c.1229del (p.Thr410MetfsX15) c.190G>A (p.Glu64Lys) c.1229del (p.Thr410MetfsX15) c.1229delC (p.Thr410MetfsTer15) c.1229delC (p.Thr410MetfsTer15) c.1607+1G>A c.1126G>T (p.Glu376*) c.2413C>T (p.Arg805*) c.2472_2478del (p.Phe825LysfsX9) c.4396C>T (p.Arg1466*) c.4495_4496insTAAT(p.Ser1499IlefsX13) c.2250G>A (p.Lys750=) c.8264_8268del (p.Tyr2755CysfsX12) c.7517_7520del (p.Arg2506ThrfsX3) c.1671_1674del (p.Ile558LysfsX2) c.2835-1G>C c.2167_2168del (p.Met723ValfsX21) c.1939C>T (p.GLN647*) c.998_999del (p.Ser333*) c.128_131delTGTT (p.Leu43TrpfsTer11) c.1723C>T (p.Gln575X) c.1427C>T (p.Thr476Met) c.6952C>T (p.arg2318*) c.1135A>T (p.Lys379*) c.743G>A (p.Arg248Gln) c.473C>T (p.Arg158His) c.251-2A>T c.1480C>T (p.Gln494Ter) c.1252del (p.Ala418ProfsX18)	Proband Cancer OC,45 BC, 31 BC, 41/ BC, 43 (contralateral) BC, 58 BC, 50 BC, 61 BC, 52 BC, 35 BC, 37 BC, 49/ BC, 64 (contralateral) Bilateral OC, 64 Asymptomatic BC, 40 Bilateral OC, 62 BC, 53 BC, 49/ BC, 52 (contralateral) BC, 66 BC, 27 BC, 72 BC, 34 BC, 76 BC, 49 BC, 48 BC, 62 ampulloma, 59/ BC, 60 Bilateral BC, 56 BC, 50/ LuC, 53 Bilateral Oc, 57 ThyC, 47/ BC, 62 BC, 56 BC, 65/ OC, 72 OC, 41/ BC, 61/ PaC, 63 Asymptomatic BC, 49	Histology Serous cystadeno carcinoma Invasive ductal carcinoma Invasive ductal carcinoma Invasive ductal carcinoma Ductal carcinoma Invasive ductal carcinoma Ductal carcinoma *In situ* ductal carcinoma Invasive ductal carcinoma Invasive ductal carcinoma Serous cystadeno carcinoma NA Invasive ductal carcinoma Serous cystadeno carcinoma Invasive ductal carcinoma Invasive ductal carcinoma Invasive lobular carcinoma Invasive lobular carcinoma Carcinoma *in situ* Invasive ductal carcinoma Invasive ductal carcinoma Unknown Carcinoma Invasive ductal carcinoma Signet-ring cell carcinoma/ BC unknown Unknown Invasive Lobular Carcinoma Serous Bordeline + Adenocarcinoma Papillary carcinoma/ Invasive ductal carcinoma Invasive ductal carcinoma Invasive ductal carcinoma/ Serous cystadeno carcinoma Adenocarcinoma/ Invasive ductal carcinoma NA Invasive ductal carcinoma	HR/HER2 status NA HR-/HER2- HR+/HER2+/HR-/HER2- HR-/HER2- Unknown HR+/HER2- Unknown NA HR+/HER2- HR-/HER+ NA NA HR+/HER2- NA HR+/HER2- HR+/HER2+/(OR+/PR-)/HER2- HR+/HER2- HR+/HER2- NA HR-/HER2+ HR+/HER2- HR+/HER2ukn HR+/HER2- HR+/HER2+ Unknown HR-/HER2- and HR+/HER2ukn HR+/HER2- NA HR-/HER2- HR+/HER2+ HR+/HER2- HR-/HER2- NA HR+/HER2-	Metastasis + - - - + - - - - - - NA + lymph node + lymph node - - - - - - - + lymph node - - - + at 57 + lymph node - - - + + NA -

BC: Breast cancer; OC: Ovarian Cancer, PaC: pancreatic cancer, LuC: lung cancer, ThyC: thyroid cancer; ukn: unknown

**Table 1B:** Family history of cancer of patients with pathogenic or probably pathogenic variants in genes other than *BRCA*

Patient number High risk gene 1 2 3 4 Moderate to low –risk gene 5 6 7 8 9 10 11 12 13 14 15 16 17 18 19 20 21 22 23 24 25 26 27 28 29 30 Mutations in two different genes in the same patient 31 32 33 Incidental Finding 34	Family History of cancer Maternal aunt: OC, 55; maternal aunt: OC, 41; maternal aunt: BC None Maternal grandfather: LuC, 70 Sister: EC, 42; mother: EC, 59; sister: BC, 51; maternal aunt: BC; maternal aunt: SyC Sister: ThyC, 54, liposarcoma, 66, BC, 68; mother: BC, 73, lymphoma, 83 Sister: OC, 54; father: LuC 70; maternal aunt: BC, 70 Mother: BC, 41, PaC, 72; maternal grandmother: BC, 64 Father: PrC, 71; paternal aunt: stomach cancer,31; mother: cerebral lymphoma, 50, BC, 74; maternal aunt: BC, 75; maternal grandfather: CRC, 74; maternal grandmother: OC, 87 Father: stomach cancer, 61 Sister: thyroid cancer, 30; maternal grandmother: CRC, 57; mother: BC, 64; maternal aunt: CRC, 62; maternal aunt: CRC, 85; maternal grandfather: PrC, 80 Sister: BC, 68; father: CRC, 71; maternal grandmother: leukemia,82 Father: PrC, 63; paternal aunt: BC, 55 and 60; paternal grandfather: PrC, 77; maternal grandmother: BC, 44; mother: BC, 63; maternal cousin: LuC, 47 Paternal uncles(2): ENT cancers, 50 Brother: systemic cancer, 48; nephew: leukemia, 6; nephew: stomach, PaC and bone cancer, 60; niece: PaC, 59; nephew: leukemia, 54; niece: BC, 50; sister: PaC, 49; niece: eiomyosarcoma, EC, brain tumor, 38; niece: EC, 32; sister: CRC, 61; niece: lymphoma, 22; brother: skin cancer, 55; mother: leukemia, 79 Father: glioblastoma, 75; paternal aunt: bilateral BC, 50; paternal cousin: BC, 50; paternal cousin: bilateral BC, 57; paternal cousin: BC, 63; paternal cousin: BC, 65; paternal cousin (male): BC, 58; paternal cousin: BC, 45; paternal uncle: spinal cord cancer, 60; mother: EC, 54, myeloma, 76; maternal grandmother: CRC, 80 Father: PrC, 67; paternal grandfather: PrC; mother: BC, 74; maternal cousin: BC, 53; maternal grandmother: stomach cancer Sister: BC, 40, bilateral BC, 70, PaC, 74; niece: BC, 32; niece: BC; niece: CeC Paternal grandfather: PrC, 60, skin cancer, 70 Twin sister: BC, 60; sister: BC, 60; nephew: PrC, 59; Father: ENT cancer, 60; mother: gynecological cancer (Œ), 41; maternal aunt: BC and bone cancer, 75; maternal aunt: SyC, 75 Mother: BC, 55; paternal grandfather: PrC, 70, liver cancer, 80 Father: LuC, 58; mother: BC, 85; maternal aunt: BC, 60 Sister: BC, 50; sister: CRC, 70; niece: brain tumor, 43; sister: BC, 49, metastatic at 59; sister: EC, 57 Sister: BC, 53, ENT, 57; paternal aunt: melanoma, 76; paternal cousin (male): bladder cancer, 58; paternal grandmother: BC, 88; paternal grandfather: liver cancer, 70; maternal uncle: LuC,79 Sister: BC, 51; father: CRC, 85; mother: BC, 75 Mother: BC, 65; maternal grandmother: BC, 48 Sister: BC, 54; sister: BC, 49 Sister: OC, 49; niece: HoD; father: PrC, 83; paternal uncle: PaC, 75; paternal cousin: BC, 42; paternal grandfather: ENT cancer; maternal uncle: PrC, 70; maternal cousin: BC, 49; maternal uncle: stomach cancer, 64; maternal cousin: BC, 42; maternal grandfather: PrC>80 Sister: BC, 43, OC, 55, metastatic at 67; father: CRC, 70; paternal cousin: BC, 55; paternal grandfather: CRC, 60 Mother: BC, 60, contralateral BC, 75, EC, 85; maternal uncle: leukemia, 71; maternal uncle: BC, 57; maternal grandmother: ENT cancer Brother: kidney cancer, 12; sister: BC, 60; sister: BC, 63; brother: brain tumor, 36; father: bladder cancer; maternal cousin: bladder cancer >60; maternal cousin: BC <50; maternal grandmother: LuC Sister: gynecological cancer, 52; niece: BC, 55; niece: BC, 60; nephew: liver cancer; mother: gynecological cancer, 52 ; maternal aunt: gynecological cancer, 50 Paternal aunt: BC Mother: BC, 34 (deceased 36); maternal grandmother: CeC, 32 Mother: OC, EC, CRC, 80; paternal grandmother: LiC, 69; maternal grandfather: LiC, 58

BC: Breast cancer; OC: Ovarian Cancer, LiC: liver cancer, GyC: gynecological cancer, SyC: systemic cancer, PaC: pancreatic cancer, CeC: cervical cancer, PrC: prostate cancer, CRC: colorectal cancer, TeC: testis cancer, HoD: Hodgkin's disease, EC: endometrial cancer, Me: melanoma, LuC: lung cancer, ThyC: thyroid cancer

### Impact of genetic testing on management

The impact of genetic testing on the management of patients is summarized in Table 2, in which patients are classified according to the presence of high-risk, moderate-low-risk, mutations in two different predisposing genes or even incidental findings. All patients were eligible for screening for a genetic predisposition according to our criteria, described in materials and methods. The identification of a mutation in a high-risk gene always led to a modification in genetic counseling and surveillance in patients with good prognosis, but also in some cases to specific risk-reduction measures (prophylactic surgery in females with MMR mutations). No treatment guidance could be counselled (avoidance of radiotherapy in females with Li-Fraumeni syndrome) since our 2 patients with TP53 mutations had metastasis and one of them died. For moderate-low-risk genes, the impact on surveillance (69%) and genetic counseling remained (89%), but there was no impact on specific-risk reduction measures or treatment guidance. These results can be attributed to the recent 2015-2016 NCCN guidelines, which recommend breast magnetic resonance imaging (MRI) screening in patients with *ATM, CHEK2* and *PALB2* mutations. The question of limiting mammography in patients with *ATM* heterozygous variants remains open to discussion as there are still discrepancies and few studies regarding the effects of radiation on those patients’ cells [32-34]. The impact on genetic counseling was often limited in this category of patients. Indeed, it is difficult to reassure negative patients, given the possibility that other HBOC predisposing genes unknown to date may be also implicated. The option of proposing breast MRI in positive patients and surveillance in negative patients according to family history was chosen. The eleven patients whose positive results after multigene panel testing had no impact on management were four women with low-penetrance *BRIP1*, *BARD1* and *RAD50* mutations, one women with *TP53* and *PALPB2* mutations who had died at time of the results, four women with a metastasis progression of her disease, one women with an *ATM* mutation who was 79 at time of the results, and one man with an *ATM* mutation. None of our patients were given customized gene-specific treatment options at the time of the study as the only example available to date (PARP inhibitors in advanced ovarian cancers) was not available at the time of the decision making. The impact was also major in patients with a mutation in two different genes, since they benefited from surveillance or specific risk-reduction measures, defined according to predispositions due to both genes, and careful genetic counseling, based on the guidelines for the two predisposing genes. Finally, a cautious approach was adopted in the individual with an incidental diagnosis of a *SUFU* mutation. One should also consider the benefit to all patients of the study in access to future research. The analysis of clinical actionability performed among individuals with mutations other than *BRCA* revealed that additional disease-specific screening and/or prevention measurers beyond those based on personal and family history alone had been recommended in more than two-third of cases, after determining whether the high-risk surveillance was maintained because of the health status, age (age cut-off for recommending breast MRI set up at 75 years in our center), or unchanged in a context of Boadicea score greater than 20 in the index case.

**Table 2:** Orientation of clinical management according to genetic information

Patient Number High risk Gene 1 2 3 4 Subtotal 1 Moderate to low-risk genes 5 6 7 8 9 10 11 12 13 14 15 16 17 18 19 20 21 22 23 24 25 26 27 28 29 30 Subtotal 2 Mutation in two different genes 31 32 33 Subtotal 3 Incidental finding 34 **Total**	Gene *TP53* *PMS2* *PMS2* *MLH1* *CHEK2* *CHEK2* *CHEK2* *CHEK2* *CHEK2* *CHEK2* *CHEK2* *CHEK2* *CHEK2* *CHEK2* *ATM* *ATM* *ATM* *ATM* *ATM* *ATM* *ATM* *ATM* *ATM* *PALB2* *PALB2* *PALB2* *BARD1* *BARD1* *BRIP1* *RAD50* *CHEK2 and BRCA2* *PALB2 and TP53* *PMS2 and BRCA1* *SUFU*	Type of cancer in proband OC,45 BC, 31 BC, 41/ BC, 43 (contralateral) BC, 58 BC, 50 BC, 61 BC, 52 BC, 35 BC, 37 BC, 49/ BC, 64 (contralateral) Bilateral OC, 64 Asymptomatic BC, 40 Bilateral OC, 62 BC, 53 BC, 49/ BC, 52 (contralateral) BC, 66 BC, 27 BC, 72 BC, 34 BC, 76 BC, 49 BC, 48 BC, 62 ampulloma, 59/ BC, 60 Bilateral BC, 56 BC, 50/ LuC, 53 Bilateral Oc, 57 ThyC, 47/ BC, 62 BC, 56 BC, 65/ OC, 72 OC, 41/ BC, 61/ PaC, 63 Asymptomatic BC, 49	Alive (A)/ Dead (D) A A A A A A A A A A A A A A A A A A A A A A A A A A A A A A A D A A	Current age of the patient 48 35 47 61 68 67 64 49 50 69 68 47 44 65 60 60 70 32 79 37 79 65 55 67 63 60 50 63 65 60 77 Died at 66 30 65	Modified surveillance (option) -(metastasis) + Lynch + Lynch + Lynch 3/4 (75%) -(metastasis) +(MRI) +(MRI) +(MRI) +(MRI) +(MRI) +(MRI) +(MRI) +(MRI) +(MRI) +(MRI, limited mammography) +(MRI, limited mammography) +(MRI, limited mammography) +(MRI, limited mammography) -(age) +(MRI, limited mammography) -(male) +(MRI, limited mammography) +(MRI, limited mammography) +(MRI) +(MRI) -(metastasis) -(no consensus) -(no consensus) -(no consensus) -(no consensus) 19/27 (70%) -(metastasis and age) - (dead) +(Lynch) 1/3 (33%) + (brain MRI) **24/35 (69%)**	Specific risk- Reduction measures -(metastasis) + + + 3/4 (75%) -(metastasis) - - - - - - - - - - - - - - - - - - - - - - - - - - - (dead) + 0/27 (0%) - **4/35 (11%)**	Treatment Guidance -(metastasis) - - - 0/4 (0%) - - - - - - - - - - - - - - - - - - - - - - - - - - - - (dead) - 0/27 (0%) - **0/35 (0%)**	Customized Treatment options - - - - 0/4 (0% - - - - - - - - - - - - - - - - - - - - - - - - - - - - (dead) - 0/27 (0%) - **0/35 (0%)**	Indentification of at-risk relatives + + + + 4/4 (100% L L L L L L L L L L L L L L L L L L L + + + - - - - + + + 24/27 (89%) + **31/35 (89%)**

BC: Breast cancer; OC: Ovarian Cancer, PaC: pancreatic cancer, LuC: lung cancer, ThyC: thyroid cancer

## DISCUSSION

Though recent studies have evaluated the positive yield of a multigene panel as compared to *BRCA1/2* testing alone, the impact of these results on patient surveillance is rarely reported. In this series, our aim was to answer this question, since it represents a central challenge for the future deployment of genomic medicine [25, 27, 35-36].

Regarding the number of pathogenic variants, our results were quite similar to those in other studies. The frequency of *BRCA1/2,* *PALB2, ATM* and *CHEK2* mutations were comparable to those in published series with the same inclusion criteria. Table 3A and 3B summarizes the primary aim of these studies, the inclusion criteria, the list of genes included in the selected panels and the results of ten published studies that included more than 500 patients who had undergone diagnostic multigene panel testing for HBOC [18-20, 22-23, 25, 27-28, 30-31]. One study was excluded from this review despite the high number of patients, because the authors chose to study only 36 known pathogenic mutations in the selected HBOC genes [14]. The highest number of patients included was 10030, most of whom had HBOC, although one study was limited to unselected TNBC patient [31] and another one to OC [29]. Two studies included exclusively patients previously tested negatively for *BRCA1/2* mutations [25, 27]. The number of genes tested varied between 8 and 27. The most recurrent genes included in the panels were *ATM, BARD1, BRIP1, CDH1, CHEK2, MLH1, MSH2, MSH6, PALB2, PSM2, RAD51C, STK11, TP53.* The overall detection rate of mutations in genes other than *BRCA* ranged from 2.9% to 9.3%. Pathogenic mutations in *CHEK2* (0-2.1%) and *ATM* (0.5-2.9%) were by far the most frequently mutated genes in this population of patients, followed by *PALB2*. NCCN guidelines for those genes have been published very recently, with some differences as compared to those for highly-penetrant genes. Indeed, breast MRI, but not prophylactic surgery, is recommended [NCCN Guideline, see URL], and caution should be exercised regarding genetic counseling in relatives. Indeed, because of the possible co-segregation of several low- or moderate-penetrance genes, surveillance in unaffected relatives not carrying the pathogenic mutation could be recommended depending on the family history. Therefore, a positive result may still be useful, but negative results may not be completely reassuring.

**Table 3A:** Other publications relating to multigene panel testing for HBOC that included more than 500 patients, including the primary aim of these studies, the inclusion criteria and the overall detection rate of pathogenic or probably pathogenic variant other than BRCA

**Reference** Tung et *al*. (18) Shirts et *al.* (19) Minion et *al*. (20) Castéra et *al.* (22) Susswein et *al*. (23) Desmond et *al*. (25) Thompson et *al*. (27) Schroeder et *al*. (28) LaDuca et *al*. (30) Couch et *al.*(31) Our study	**Number of patient** Cohort 1, n= 1781 Cohort 2, n=377 Patients with prior negative (BRCA1/2 test) 1462 refered for tetsting by BROCA (1066) or ColoSeq (396) multigene panels 911 708 10030, including 5209 with female BC, 51 male BC patients and 845 OC 1069 2000 cases and 1997 controls 620 2079 including 874 cases from BC panel and 223 cases from OC panel 1824 583	**Aim of the study** To assess the frequency of deleterious germline mutations using a panel of 23 genes associated with inherited cancer predisposition To evaluate multigene panels for inherited cancer predisposition in a clinical series that included multiple cancer types and complex families histories To investigate the contribution of 19 predisposing genes for HBOC To prospectively evaluate the performance of NGS for routine analysis of *BRCA1* and *BRCA2* and to determine the rate of deleterious mutations within other genes in a large series of HBOC patients To report the experience of well-established high-risk cancer genes as well as more recently identified predisposing cancer genes (29 multigene panel) To define the potential clinical effect of multigene panel testing To assess the frequency of mutations in 18 genes included in hereditary BC panels among index cases and matched population controls To study the mutation detection rate using diagnostic HBOC multigene panel in two centers of the German consortium To identify inherited risk for OC to allow effective and targeted prevention To assess the frequency of mutations in 17 predispositions genes, including *BRCA1* and *BRCA2* to determine the utility of germline genetic testing in triple negative breast cancer (TNBC) To determine the clinical actionability of a multigene panel testing for HBOC	**Selection criteria** Patients referred for *BRCA1/2* testing 1462 sequential patients referred for testing of germline DNA with the BROCA or ColoSeq panel *BRCA1* and *BRCA2* negative population of patients with a personal history of BC and/or OC Patients seen in the context of a genetic consultation and fulfilled at least one of the criteria defined by the author (see methods for more information) Clinical genetic testing performed for evaluation of germline cancer genes Candidates for HBOC evaluation and who lacked *BRCA1/2* mutations Women mainly affected with BC with strong family history of BC and negative BRCA1 and 2 screening. Controls were cancer-free women from the LifePool study (see reference) Indication for HBOC testing according to the GC-HBOC 273 women with OC, 48 with peritoneal carcinomas, 31 with fallopian tube carcinomas, and 8 with synchronous endometrial and OC. Patient with TNBC unselected for family history of breast or ovarian cancer Sporadic BC before 40 ; sporadic OC before 70 ; sporadic BC in a male at any age ; at least two BCs amongst which one before 50 ; at least two BCs amongst which one OC at any age ; at least three BC at any age	**Overall detection rate of pathogenic or probably pathogenic variants other than *BRCA*** Cohort 1: 4.4% Cohort 2: 3.7% 6.9% for BC patients 7.1% for OC patients 7.4% 6.6% 9.3% 5.9% 3.9% for cases and 1.6% for controls 2.9% 7.4% for BC panel and 7.2% for OC panel 3.7% 6.8%

HBOC: hereditary Breast and ovarian Cancer; BC: breast cancer; OC: ovarian Cancer

**Table 3B:** Genes included in the selected panels and their positive yield (number of pathogenic or likely pathogenic variants)

**Reference** Tung et *al*. (18) Shirts et *al.* (19) Minion et *al*. (20) Castéra et *al.* (22) Susswein et *al*. (23) Desmond et *al*. (25) Thompson et *al*. (27) Schroeder et *al*.(28) LaDuca et *al*. (30) Couch et *al.*(31) Our study	Genes other than BRCA1 and 2* included in the panel and percentage of pathogenic or probably pathogenic mutations found for each gene ***TP53** (**Cohort1/0.1**; Cohort2/0), **CDH1** (C1/0; **C2/0.5**), PTEN (C1/0; C2/0), **ATM (C1/0.7; C2/0.3)**, **CHEK2 (C1/1.6; C2/1.3)**, STK11 (C1/0; C2/0), RAD51C (C1/0; C2/0), **PALB2 (C1/0.7; C2/0.3)**, **BARD1 (C1/0.3; C2/0.3)**, **BRIP1 (C1/0.4**; C2/0), **NBN (C1/0.2); C2/0.3)**, MLH1 (C1/0; C2/0), **MSH2 (C1/0.06**; C2/0), **MSH6 (C1/0.1**; C2/0), **PMS2 (C1/0.2**; C2/0), EPCAM (C1/0; C2/0), RAD51D (C1/0; C2/0), **APC** (C1/0; **C2/0.3**), **MUTYH (C1/0.1; C2/0.3)**, CDKN2A (C1/0; C2/0), SMAD4 (C1/0; C2/0), CDK4, (C1/0; C2/0) BMPR1A (C1/0; C2/0)* **BROCA: *BRIP1 (0.6/0.9),*** *BARD1(0/0)**, CHEK2 (2.3/0.9),** MRE11A (0/0)**, NBN (0.2/0.9),** RAD50 (0/0)**,** RAD51C (0/0)**, PALB2 (1.7/1.8), TP3 (0.6/0), PTEN (0.2/0),** STK11 (0/0)**,** CDH1 (0/0)**, ATM (1.3/0.9),** MLH1 (0/0)**, MSH2 (0/0.9), MSH6 (0/0),** PMS1 (0/0)**, PMS2 (0/0.9),** MUTYH (0/0)* ***ATM (0.8), BARD1 (0.2), BRIP1 (1.1), CDH1 (0.1), CHEK2 (1.8), EPCAM (0), MLH1 (0.2), MRE11A (0.1), MSH2 (0), MSH6 (1.1), MUTYH (0), NBN (0.4), PALB2 (0.4), PMS2 (0.1), PTEN (0.2), RAD50 (0.2), RAD51C (0.3), STK11 (0), TP53 (0.3)*** ***ATM (0.8)**, BAP1 (0), **BARD1 (0.1)**, BRIP1 (0), **CDH1 (0.1)**, **CHEK2 (0.7)**, MLH1 (0), **MLH3 (0.3)**, **MRE11A (0.6)**, **MSH2 (0.6)**, MSH6 (0), NBS1 (0), **NBN (0.7)**, **PALB2 (0.8)**, **PMS1 (0.1)**, **PMS2 (0.3)**, PTEN (0), **RAD50 (0.3)**, RAD51 (0), **RAD51B (0.1)**, **RAD51C (0.4)**, RAD51D (0), STK11 (0), **TP53 (0.6)**, XRCC2 (0), XRCC3 (0)* ***MSH6 (0.3), PMS (0.2), MLH1 (0.5), MSH2 (0.4), TP53 (0.2), PTEN (0.1), MUTYH (0.2), APC (0.1) VHL (0.01), CHEK2 (1.6), ATM (0.8), PALB2 (0.5), BRIP1 (0.2), FANCC (0.2), NBN (0.1), BARD1 (0.1), RAD51C (0.1), RAD51D (0.1), XRCC2 (0.3), AXIN2 (0.01),** BMPR1A (0/0/0), CDH1 (0/0/0), CDK2NA (0/0/0, EPCAM (0/0/0), SMAD4 (0/0/0), STK11 (0/0/0), CDK4 (0/0/0)* ***CDH1 (0.4), TP53 (0.3), CHEK2 (1.4), ATM (1.0), BARD1 (0.1), PALB2 (0.8), RAD51C (0.3), NBN (0.2), BRIP1 (0.1), PMS2 (0.4), MSH2 (0.2), MSH6 (0.2), MLH1 (0.1), CDKN2A (0.3), APC (0.1), BMPR1A (0.1), MUTYH (0.2)*** ***ATM (0.4/0.2), ATR (0.1/0.05), BARD1 (0.1/0.05), BLM (0.2/0.15), BRIP1 (0.3/0.20), CDH1 (0.05/0), CHEK2 (0.4/0.3), MRE11A (0.2/0), NBN (0.1/0.1), NF1 (0.05/0.05), PALB2 (1.3/0.2), PTEN (0.05/0), RAD50 (0.1/0.3)**, STK11 (0/0), **TP53 (0.2/0), XRCC2 (0.1/0)*** ***ATM (0.7), CDH1 (0.5), CHEK2 (1.0), NBN (0.3), PALB2 (0.3)**, RAD51C (0), RAD51D (0), **TP53 (0.2)*** **Breast panel: *ATM (2.1),*** *BARD1 (0)**, BRIP1 (0.1),** CDH1 (0)**, CHEK2 (2.2), MRE11A (0.1),** MUTYH (0)**, NBN (0.1), PALB2 (1.7), PTEN (0.3), RAD50 (0.3), RAD51C (0.2),** STK11 (0), **TP53 (0.5)*** **Ovarian Panel: *ATM (0.9),*** *BARD1 (0), **BRIP1 (0.9),CDH1 (0.4), CHEK2 (0.4**), EPCAM (0)**,** MLH1 (0)**, MRE11A (0.4),** MSH2 (0), **MSH6 (1.3),** MUTYH (0), **NBN (1.3), PALB2 (0.4), PMS2 (0.4),** PTEN (0), RAD50 (0), **RAD51C (0.4),** STK11(0), **TP53 (0.4)*** ***PALB2 (0.1)**, **BARD1 (0.5)**, **BRIP1 (0.4)**, **RAD51C (2.2)**, **RAD51D (0.3)**, **RAD50 (0.3),NBN (0.05)**, **MRE11A (0.1)**, **XRCC2 (0.2)**, **ATM (1.2),** CHEK2 (0), **TP53 (0.05),PTEN (0.05)**, STK11 (0), CDH1 (0)* *APC (0), **ATM (1.5)**, **BARD1 (0.3)**, **BRIP1 (0.2)**, CDH1 (0), **CHEK2 (2.1)**, EPCAM (0), **MLH1 (0.2)**, MRE11A (0), MSH2 (0), MSH6 (0), MUTYH (0), **PALB2 (0.7)**, PIK3CA(0), **PMS2 (0.5)**, PTCH1 (0), PTCH2 (0), PTEN (0), **RAD50 (0.2)**, RAD51C (0), STK11 (0), **SUFU (0.2)**, **TP53 (0.5)***

*MUTYH monoallelic pathogenic mutations were not taken into account

Interestingly, some high-risk genes were detected in our series as in others. We identified two patients carrying three mutations in *TP53.* Neither of our two patients met the Chompret criteria (Figure 2). Detecting these mutations is extremely important for screening and genetic counseling NCCN guidelines [see URL], including the avoidance of irradiation in the treatment of cancer whenever possible, in order to decrease the risk of a second malignancy. We also identified four mutations in MMR genes (*PMS2* and *MLH1*), but in three of our four families, there were exclusively breast cancer pedigrees (Figure 2). With the experience of gene panels, which included HBOC susceptibility genes among others, some families harboring a mutation in an MMR gene did not meet Amsterdam II or Bethesda criteria for Lynch syndrome testing [37]. To date, it is unclear whether the results in our three families with single-affected early-onset BC should be regarded as incidental or not since an increased risk of BC in Lynch syndrome is still controversial [38-40]. Nevertheless, massively-parallel sequencing targeting multiple candidate genes is changing the paradigm as it gives the opportunity to assess the role of genes that would never have been evaluated in a non-panel approach. Regarding this point, our study suggests that *PMS2*, which does not contribute greatly to Lynch Syndrome [41], could be considered a good candidate for evaluation. Moreover, as the commonly known technical difficulties of analyzing *PMS2* are slowly being overcome by new analysis strategies [41-42], critically needed evidence concerning the role of *PMS2* can be expected. For instance, recently, ten Broeke et *al.* reported a standardized incidence ratio of 3.8 for breast carcinomas, which led them to suggest adding mammography from age 40 years in *PMS2* families with evident clusters of BC [43]. Finally, one mutation was found in *SUFU*, and considered an incidental finding given the absence of a personal or family history of medulloblastoma or Gorlin syndrome.

One of the greatest advantages of gene panels compared with testing single genes is the possibility of diagnosing patients with a mutation in more than one predisposing gene, thus leading to more accurate genetic counseling. Indeed, one of our index cases had two *TP53* mutations on the same allele and a *PALB2* mutation. Detection of the *TP53* mutations alone would have led to inaccurate genetic counseling since negative patients for the *TP53* mutations would have been reassured whereas adapted breast screening is justified in *PALB2* carriers. The same situation exists for the family with the index case carrying a *BRCA* and a *CHEK2* mutation. Also, the detection of a *PMS2* mutation in an index case with a *BRCA* mutation led to the prescription of digestive and pelvic follow-up.

In this work, we aimed to assess how and whether these advances in technology will be applied in clinical practice, and how best to counsel patients about variants of low or moderate penetrance. Taking advantage of a single clinical genetics team and laboratory in an entire region of France (Burgundy), we had the opportunity to follow the clinical value of multigene testing, especially in the context of recently updated practice guidelines from the USA for moderate-risk HBOC genes. We first identified six individuals with mutations in high-risk genes, associated or not with the clinical presentation of the family, which had been missed by the traditional focused approach to testing. In these cases, the results changed the management of the patients and the at-risk family members, thus justifying the testing of additional family members in all cases. The most challenging results were found in moderate- to low-penetrance HBOC genes. When we applied gene-based consensus practice guidelines when they existed, we found that the sequencing results modified management in the majority of individuals (70%) and prompted testing in the majority of families (89%). In the future, therapeutic options might be evaluated in members of families carrying a mutation in a DNA-repair gene who eventually develop cancer, such as PARP-inhibitor strategies [US FDA breakthrough therapy designation see URL, 44]. Importantly, family members who tested negative for the familial mutation were not reassured, and were managed according to empiric risk depending on the patient's personal and family history of cancer.

The choice of following the NCCN guidelines as a basis for assessing the impact of panel testing is the main limitation of the work. Indeed, the NCCN has proposed high-risk surveillance including MRI in *ATM* and *CHEK2* mutation carriers, which represent the majority of mutations identified by the panel in predisposing genes other than *BRCA*. Other authors have warned about the risk of disinformation and the implementation of inappropriate risk-management strategies in cases with limited cancer risk management guidelines, as such guidelines are not sufficiently developed to allow accurate targeted genetic counseling and breast cancer risk management [27]. However, the maintenance of surveillance in non-carrier relatives should limit this risk. Another limitation of the study is the possible overestimation of the percentage of clinically actionable mutations since there is no consensus in France regarding an age cut-off for recommending breast MRI.

In conclusion, multigene panels, which include cancer susceptibility genes that would have escaped clinical investigation because the familial presentation did not meet the adopted criteria for testing, make it possible to identify more individuals with a genetic predisposition for HBOC. Such a strategy, rather than *BRCA* testing alone, will modify the management of such patients. This strategy also identifies individuals with multiple-gene predispositions, which should lead to caution in genetic counseling. The classification of patients according to their predisposing genes will be of increasing importance with the arrival of customized treatment options. Given the complex issues raised by multi-gene panel analysis, pre- and post-test genetic counseling and informed decision making are of major importance. As increasing data from multigene panel testing become available, we anticipate that international research efforts will lead to more accurate risk estimates and a better classification of cancer spectra and genetic variants. This in turn will drive the development of explicit and specific management guidelines. However, it will also increase the level of uncertainty since massively-parallel sequencing targeting multiple candidate genes will greatly increase the number of patients exhibiting VUS in different candidate genes.

## MATERIALS AND METHODS

### Patients

The study involved the first 583 consecutive patients from Burgundy (France) to benefit from diagnostic panel testing for HBOC, from November 2012 to November 2013 at one of the five genetic clinics of Burgundy. All of the patients, who met the criteria for *BRCA1* and *BRCA2* testing, had pre- and post-test genetic counseling by a geneticist and/or genetic counselor: i) personal history of single-affected BC before age 40; ii) personal history of single-affected OC before age 70; iii) personal history of single-affected male BC before age 70; iv) personal history of single-affected triple-negative BC before age 50; v) two BC in first or second-degree relatives with at least one cancer before age 50; vi) one BC before age 50 with pancreatic or prostate cancer before age 60 in first or second-degree relatives; vii) three BC in first or second-degree relatives at any age. Testing in first-degree relatives unaffected by cancer of an individual with a very suggestive family history of HBOC was also proposed if it was impossible to test the relatives affected with cancer. Patients had not undergone specific *BRCA* testing before the multigene panel analysis. For each patient, informed consent for genetic analysis was obtained. General information was entered into the Oncogene database, an in-house database generated via Clinsight, used for the management of Oncogenetic consultations and is registered with the Commission Nationale Information et Liberté (CNIL). This database includes demographic data, self-identified personal and family histories of cancer, and follow-up for patients with a pathogenic or probably pathogenic mutation in a gene predisposing for HBOC.

### Sample preparation and next-generation sequencing

DNA was extracted from peripheral blood, using a Maxwell BioRobot system (Promega, Madison, USA). 3 μg of DNA was sonicated using a Covaris S2 (Covaris, Inc, MS, Woburn, MA, USA) and purified. Following capture, samples were barcoded with 16 different indexed primers, pooled per lane and sequenced. The Agilent eArray was used to design the SurSelect solution library (Agilent, Santa Clara, CA, USA) covering a panel of 25 genes including 20 genes with a predisposition for breast and/or ovarian cancer: *BRCA1, BRCA2, BARD1, CHEK2, RAD51C, BRIP1, RAD50, MRE11A, PALB2, STK11, MLH1, MSH2, MSH6, EPCAM, PMS2, PTCH1, PTCH2, CDH1, TP53, ATM, PTEN, PIK3CA, SUFU, MUTYH* and *APC*. This design was kindly provided by Nicolas SEVENET's laboratory (Institut Bergonié, Bordeaux, France) and was developed to meet their diagnostic needs for HBOC, predisposition to digestive cancer or polyposis, and Basal Cell Naevus (Gorlin) syndrome. These genes were not excluded from the bioinformatics analysis since keeping them could have raised the question of incidental findings, which could be important to address for our future practice. The panel did not include all HBOC genes. *NBN, NF1, RAD51D* and *XRCC2* were missing as the panel was designed before the interest of these genes became apparent.

Exon capture was limited to the 451 exonic sequences of the 25 genes with 50 bp surrounding intronic sequences on each side of the exon. The total size of the library was 192 Kb. The library was sequenced on Miseq (Illumina, San Diego, CA, USA) using the paired-end 2×150 bp program.

### Large rearrangement analysis for *BRCA1* and *BRCA2* genes

Large rearrangements were identified by profile comparisons, using the semi-quantitative MLPA method (MRC-Holland, Amsterdam, Netherlands). Briefly, electropherograms from patients were first superposed, the yield of each amplicon in the various samples was evaluated and deletions/duplications of one or more amplicons were revealed by a 2-fold decrease/1.5-fold increase in the corresponding peak, respectively [45]. Large rearrangements were not tested for the other genes of the panel.

### Bioinformatics analysis

An average of 215 million high-quality bases were generated for each sample. Raw reads from MiSeq were first aligned to the human genome reference GRCh37/hg19 from UCSC Genome Browser with the Burrows-Wheeler Aligner (BWA, v0.7.6a) [46]. Duplicate paired-end reads were marked with Picard v.1.109. Base quality score recalibration, realignment around indels, and variant discovery were performed with the Genome Analysis Toolkit (GATK) v3.3-0 [47]. Detected variants were annotated with the SeattleSeq annotation portal according to the mutation type, the occurrence in dbSNP and the NHLBI Exome Sequencing Project Exome Variant Server [see URL, 48].

Candidate events were systematically identified by focusing on protein-altering and splice-site variants present at a frequency less than 1% in dbSNP 141, and supported by at least three reads in the subject at base-pair positions covered by at least five reads.

### Variant classification

Sequence variants and large insertions and deletions were classified according to the American College of Medical Genetics (ACMG) guidelines for variant interpretation [49]. Variants were classified as pathogenic or probably pathogenic if they conferred a truncating, initiation codon or splice donor/acceptor effect, if functional data demonstrated an effect on protein function relevant to a disease phenotype, or if pathogenicity was otherwise demonstrated in the published literature. If no functional data were available, missense, silent, and intronic variants were classified as variants of uncertain significance (VUS), benign or probably benign based on allele frequency in dbSNP or the Exome Variant Server [see URL]. In the genes other than *BRCA*, a variant was considered for review when it was classified as pathogenic, probably pathogenic or discordant according to ClinVar. The review was performed by a multidisciplinary team and based on data in the literature. The tools for variant classification included different software and databases (mutation taster, Align GVG Database, BRCA share, ClinVar, NHGRI BIC, ExAc and LOVD BRCA) [see URLs].

### Confirmation of the detected variations

For *BRCA1* and *BRCA2*, all the variants detected by NGS within the coding sequence or ± 50bp within the intronic sequences and not recorded as polymorphisms were confirmed by Sanger sequencing, using the BigDye Terminator Cycle Sequencing V1.1 Ready reaction Kit (Life Technologies, Carsbad, CA, USA). For the other genes included in the capture enrichment, all variations inducing a premature stop codon or classified as pathogenic or probably pathogenic were checked by Sanger sequencing.

### Data analysis

The incidence of pathogenic and probably pathogenic mutations was calculated for each gene, and the clinical presentations were discussed based on demographic information and clinical history. Genes were grouped in four categories: highly penetrant genes (*BRCA1, BRCA2, STK11, MLH1, MSH2, MSH6, EPCAM, PMS2, CDH1, TP53, PTEN*), moderately penetrant/HR pathway (*BARD1, CHEK2, RAD51C, BRIP1, RAD50, MRE11A, PALB2, ATM*), one candidate gene for HBOC (PIK3CA) and other cancer-spectrum predisposing genes (*APC, MUTYH, PTCH1, PTCH2, SUFU*). Pathogenic mutations in the latter category, in the absence of cancer within the disease spectrum known for these genes in the patient or family members, were considered incidental findings. The impact of having two predisposing genes was particularly studied. Monoallelic *MUTYH* mutation carriers were not included in the mutation-positive cohort. The incidence of VUS was only given for the cohort overall.

In order to determine whether the discovery of a genetic susceptibility for HBOC guided the clinical management, we stratified the implications for patients according to five options: i) modification of cancer surveillance; ii) suggestion of specific risk-reduction measures, such as prophylactic surgery; iii) offering treatment guidance, such as avoidance of radiotherapy in TP53 patients; iv) providing customized gene-specific treatment options, such as PARP inhibitors in *BRCA* patients; v) identification of at-risk family members. The possibility of access to research projects was also considered a significant option. Each option was discussed in interdisciplinary meetings, according to the review of the published literature and practice guidelines. Patients with *BRCA* mutations were excluded from this part of the analysis because such testing is the traditional standard of care, and the aim of this study was to assess the impact on management of testing for any genes beyond the traditional single-gene approach. Since we could expect that, in some cases, the health status of affected patients may not warrant changes to their management (in particular in patients with ovarian cancer), and in other cases, patients would have been candidates for intensive screening anyway because of their very high-risk family history (remaining risk for breast cancer greater than 20%), the effective changes in their medical management guidelines were reviewed case by case.

### URL

National Comprehensive Cancer Network (NCCN). Genetic/Familial High-Risk Assessment: Breast and Ovarian. NCCN Clinical Practice Guidelines in Oncology. Fort Washington, PA: NCCN https://www.nccn.org/professionals/physician_gls/f_guidelines_nojava.asp#detection

Lynparza^TM^ (olaparib) granted breakthrough therapy designation by US FDA for treatment of BRCA1/2 or ATM gene mutated metastatic castration resistant prostate cancer http://www.sec.gov/Archives/edgar/data/901832/000119163816001590/azn201601286k.htm

Exome Variant Server, NHLBI GO Exome Sequencing Project (ESP), Seattle, WA http://evs.gs.washington.edu/EVS/

NHS guidelines https://www.nice.org.uk/guidance/cg164/chapter/1-recommendations#surveillance-and-strategies-for-early-detection-of-breast-cancer

dbSNP http://www.ncbi.nlm.nih.gov/projects/SNP/

Exome Variant Server http://evs.gs.washington.edu/EVS/

mutation taster http://www.mutationtaster.org/

Align GVG Database http://agvgd.iarc.fr/

BRCA Share http://www.umd.be/BRCA1/

ClinVar http://www.ncbi.nlm.nih.gov/clinvar/

NHGRI BIC https://research.nhgri.nih.gov/bic/

ExAc http://exac.broadinstitute.org/

LOVD BRCA http://chromium.lovd.nl/LOVD2/cancer/home.php?select_db=BRCA1
